# Effect of robot-assisted training on cognitive function in post-stroke patients: a meta-analysis

**DOI:** 10.3389/fneur.2026.1725457

**Published:** 2026-03-03

**Authors:** Juan Wang, Man Ding, Chao Weng, Hui Cai

**Affiliations:** Department of Neurology, Renmin Hospital of Wuhan University, Wuhan, Hubei, China

**Keywords:** cognitive function, meta-analysis, RCT, robot-assisted training, stroke

## Abstract

**Background:**

About 1/3 of stroke patients worldwide experience post-stroke cognitive impairment (PSCI). The management of cognitive function (CF) after stroke is an important issue that needs to be addressed. In recent years, robot-assisted training (RAT) has been widely used in the rehabilitation of CF, its intervention effect is still controversial. Therefore, this study was aimed at reporting the latest meta-analysis (MA) and evidence updates to compare the effects of RAT with traditional training (TT) on CF in post-stroke (PS) patients.

**Methods:**

Databases (PubMed, Embase, Cochrane Library and Web of Science) were retrieved to include randomized controlled trial articles that met the criteria. The intervention group used RAT, and the control group used TT. Outcome measures mainly included the Montreal Cognitive Assessment score (MoCA), and so on. Study screening, quality assessment, and data extraction were done separately by two investigators. The stability of results and potential sources of heterogeneity were explored by sensitivity and subgroup analyses. Data were pooled by RevMan 5.4 and STATA 15.0.

**Results:**

A total of 13 studies with 488 patients were included. The MA results showed that in PS patients, RAT significantly improved the MoCA score [SMD = 0.43, 95% CI (0.04–0.81), *p* = 0.03]. The sensitivity analysis showed significant instability in the changes in MoCA score and change in Frontal Assessment Battery (FAB) score. Therefore, the effect of RAT on CF in PS patients should be interpreted with caution.

**Conclusion:**

RAT improved CF in PS patients to some extent. However, evidence for this conclusion was of low quality. Therefore, further studies are still required for confirmation.

**Systematic review registration:**

https://www.crd.york.ac.uk/PROSPERO, identifier (CRD42024568846).

## 1 Introduction

Cerebrovascular disease is a general term for brain diseases caused by cerebrovascular lesions or blood flow disorders due to various factors. Common acute cerebrovascular diseases include cerebral infarction, cerebral hemorrhage, subarachnoid hemorrhage, and intracranial venous thrombosis. Their incidence, recurrence, mortality, and disability rates are high, imposing a heavy burden on patients, families, and society (1). In addition, motor impairment and cognitive impairment caused by stroke significantly affect the quality of life (QoL) in patients and their families (2–5). Globally, about 1/3 of stroke patients experience PSCI (6), a clinical syndrome characterized by cognitive impairment, which occurs after a stroke event and lasts up to 6 months (7). As an important cause of the current burden of stroke, this syndrome seriously affects the QoL and survival time, and has become a hot topic of international research on stroke and a focus of clinical intervention nowadays. Motor performance has been shown to be linked to overall CF, memory, and executive function, and cognitive impairment may weaken the restoration of activity after stroke (8–10). In addition, PSCI may result in an increase in disability rates, caregiving stress, and disease burden on families and the society, as well as a significant decrease in QoL, the ability to perform activities of daily living (ADL), and mental health status in patients. Early identification of PSCI is crucial for initiating early cognitive rehabilitation. Therefore, it is urgent to address the management of CF in PS patients.

Overall CF, and particularly attention, memory, and visuospatial ability, can be improved maximally at PS 4 months (11). It is therefore important to choose the most appropriate and effective strategy for rehabilitation in the acute PS period. In recent years, robot-assisted rehabilitation techniques have been more and more widely used in clinical practice, as the application of artificial intelligence and computer technology in the field of rehabilitation medicine continues to deepen. Robot-assisted therapy is a new approach to providing safe, repetitive, intensive, and quantitative rehabilitation interventions (12). Robots can precisely control interactions with users (e.g., support or resistance in a desired assistive manner) and visually and mechanically present virtual environments; hence, they are desirable tools for sensory motor training to provide engaging and challenging therapy (13, 14). In the past 20 years, multiple robotic devices for training the proximal upper limb (15) have been developed and clinically assessed; such devices achieved outcomes equivalent to those achieved by dose-matched traditional therapy (13, 14, 16–22). Rodgers et al. (22) performed a multicenter randomized controlled trial (RCT) to evaluate the clinical efficacy of RAT using the MIT-Manus robotic gym system, compared with an enhanced upper limb therapy program based on repetitive functional task practice (RFTP) and usual care. Their results showed no differences in upper extremity function among PS patients who used the system, program for RAT, or usual care. Torrisi et al. (23) conducted an RCT using the device Amadeo™ for upper limb functional training, which notably used 2D virtual reality (VR)-based feedback. Their results showed that both motor function in the paretic arm and global/specific cognitive abilities were enhanced by task-oriented VR-based robotic rehabilitation in PS patients. Taravati et al. (24) performed an RCT using ReoGo™-Motorika (an upper extremity rehabilitation system), in which the intervention group received 30-45 min of robot-assisted therapy, 5d weekly for 4 wk. This RCT showed an improved MoCA score in each group, but no intergroup difference. They suggest that it was not possible to assess whether improvement occurred during a long period of time, because the beneficial effect of robotic rehabilitation on cognitive problems may be too small, and only the results of a program for 4-week rehabilitation were presented.

As shown in a MA by Moucheboeuf et al. (25) regarding the effects of robotic gait training after stroke, robot-assisted gait training plus physiotherapy and body-weight support training seems to be an efficient intervention for post-stroke gait recovery. In their systematic reviews and MA regarding the efficacy of RAT in recovery of upper limb function in stroke patients, Chien et al. (26) and Yang et al. (27) suggest that RAT for upper limbs can significantly improve upper limb motor function and ADL performance in stroke patients. In addition, in a MA by Lin et al. (28) regarding the effectiveness of virtual reality games in improving cognition, mobility, and emotion in elderly PS patients, the author suggests that virtual reality games were more effective in improving overall CF in stroke patients compared to conventional therapies, and virtual reality games can effectively relieve depression and improve mental health in stroke patients. A MA by Chen et al. (29) indicates that VR training is able to improve CF and ADL performance in PSCI patients.

RAT uses multimodal sensing (such as visual, auditory, physiological, and neuro-sensing) to monitor patients’ conditions in real-time, and employs artificial intelligence for personalized adaptive training. Through techniques like gamification and virtual reality, patient engagement is enhanced. Multimodal feedback (such as visual, auditory, and tactile) is utilized to strengthen interaction and learning outcomes. Human-robot collaboration and social cognition are harnessed to facilitate natural interaction, thereby improving specific cognitive domain functions such as social communication, memory, and attention (30). Kim et al. (31) reported that RAT can induce structural and activity changes in brain areas related to cognitive function (such as the anterior cingulate cortex) through neuroimaging techniques (such as functional magnetic resonance imaging, fMRI), suggesting it may enhance the function of neural circuits via task-specific training. The underlying principle may be that, by repeatedly activating the target circuits, training solidifies specific neural activity patterns, strengthens synaptic efficacy between related neurons, and ultimately enhances the information processing capability of the entire neural circuit.

Overall, systematic reviews of the effect of RAT in PS patients are mostly aimed at analyzing upper- and lower-limb motor function and balance function. Research on the effect on CF has focused more on VR training. There are few systematic reviews of the effect on CF after stroke. Additionally, although RAT has been used frequently in PS motor and cognitive rehabilitation, its effect is still controversial. Therefore, in this study, a MA was conducted of the effect of RAT in PS patients with cognitive dysfunction to provide evidence for its use in clinical settings.

## 2 Materials and methods

This study was completed as per the PRISMA statement and already registered at https://www.crd.york.ac.uk/PROSPERO (CRD 42024568846).

### 2.1 Inclusion criteria

#### 2.1.1 Study type

An RCT to investigate the effect of RAT on CF in PS patients.

#### 2.1.2 Study subjects

Subjects with stroke confirmed by relevant imaging examinations, who meet the 1989 WHO diagnostic criteria for stroke or the diagnostic criteria established by the 1995 Fourth National Conference on Cerebrovascular Disease. Inclusion criteria: (1) ≥18 years, and within 6 months after disease onset; (2) a certain degree of cognitive ability, only mild or moderate cognitive impairment (such as a MoCA score of 10–25), basically able to cooperate on the completion of training; (3) stable vital signs.

#### 2.1.3 Interventions

The control group was given traditional rehabilitation therapy. The intervention group was treated with RAT based on intervention for the control group. The specific robot equipment models used in the studies, intervention frequency, duration of each training session, total intervention cycle, and key elements of the training content are shown in Supplementary File S1.

#### 2.1.4 Primary outcome measure

Cognitive function assessment: Montreal Cognitive Assessment (MoCA); Weigl test, and Frontal Assessment Battery (FAB); Mini-Mental-State-Examination (MMSE), Attentive Matrices (AM), Ray Auditory Verbal Learning Test (RAVLT), Trail Making Test Form (TMT), Corsi Block Tapping Test Forward (Corsi F), Corsi Block Tapping Test Backward (Corsi B), Weigl Test (WEIGL), etc.

#### 2.1.5 Secondary outcome measures

(1) Psychological assessment: the Hamilton Rating Scale for Depression (HAM-D), Hamilton Rating Scale for Anxiety (HAM-A), Beck Depression Inventory-II (BDI-II), Short Form-12 Health Survey Mental Health (SF-12 MH), etc.; (2) Motor function assessment: Functional Independence Measure (FIM), Fugl-Meyer Assessment (FMA), Functional Ambulation Classification (FAC), etc.

### 2.2 Exclusion criteria

(1) Conference papers; (2) incomplete data that cause failure to extract valid outcome data from articles; (3) duplicates; (4) designs for methodological studies, systematic reviews, etc.

### 2.3 Search strategy for studies

Databases (PubMed, Cochrane Library, Web of Science, Embase) were searched using a computer, with a search time frame until May 2025. The literature search was conducted using subject headings + free-text terms. Search terms and free-text terms included robotic, robot, robot-assisted, cognitive function, and stroke. Detailed search strategy is provided in Supplementary File S2.

### 2.4 Study screening and data extraction

All retrieved studies were entered into EndNote to remove duplicates. The remaining studies were initially screened by two investigators regarding their titles and abstracts. The preliminarily eligible studies were re-screened based on full texts according to the inclusion criteria. Relevant data were extracted from the included studies. Extracted data mainly contained the author, year of publication, country, study period, age, gender, follow-up period, and number of cases investigated.

### 2.5 Quality assessment of included studies

The quality of included studies was assessed using the Cochrane risk of bias (RoB 1.0) tool. The tool contains random sequence generation, allocation concealment, blinding of investigators and participants, blinding of outcome assessors, completeness of outcome data, selective reporting of study results, and other sources of bias. Each item was assessed by the investigator as having a low, high, or unclear risk of bias.

### 2.6 Statistical methods

RevMan 5.4 and STATA 15.0 were employed for data analysis. Measurement data were pooled using the mean difference (MD) or standardized mean difference (SMD) and 95% confidence interval (CI). Categorical variables were pooled using the risk ratio (RR) and 95% CI. Heterogeneity among the studies was assessed using the Cochran’s Q test and *I*^2^. *I*^2^ ≤ 50% or *p* ≧ 0.1 indicated low heterogeneity. Because clinical heterogeneity in patient populations and intervention details was taken into account in advance, the random-effects model was chosen, as it can take into account both intra- and inter-study variations. In addition, the stability of results and potential sources of heterogeneity were assessed by sensitivity analysis and subgroup analysis. Publication bias was assessed by the funnel plot and Egger’s test. *p* < 0.05 indicated a statistically significant difference. Additionally, according to GRADE, each outcome’s evidence was evaluated and graded as “high,” “moderate,” “low,” or “very low” quality to draw conclusions (32).

## 3 Results

### 3.1 Search results for studies

**Figure 1:**
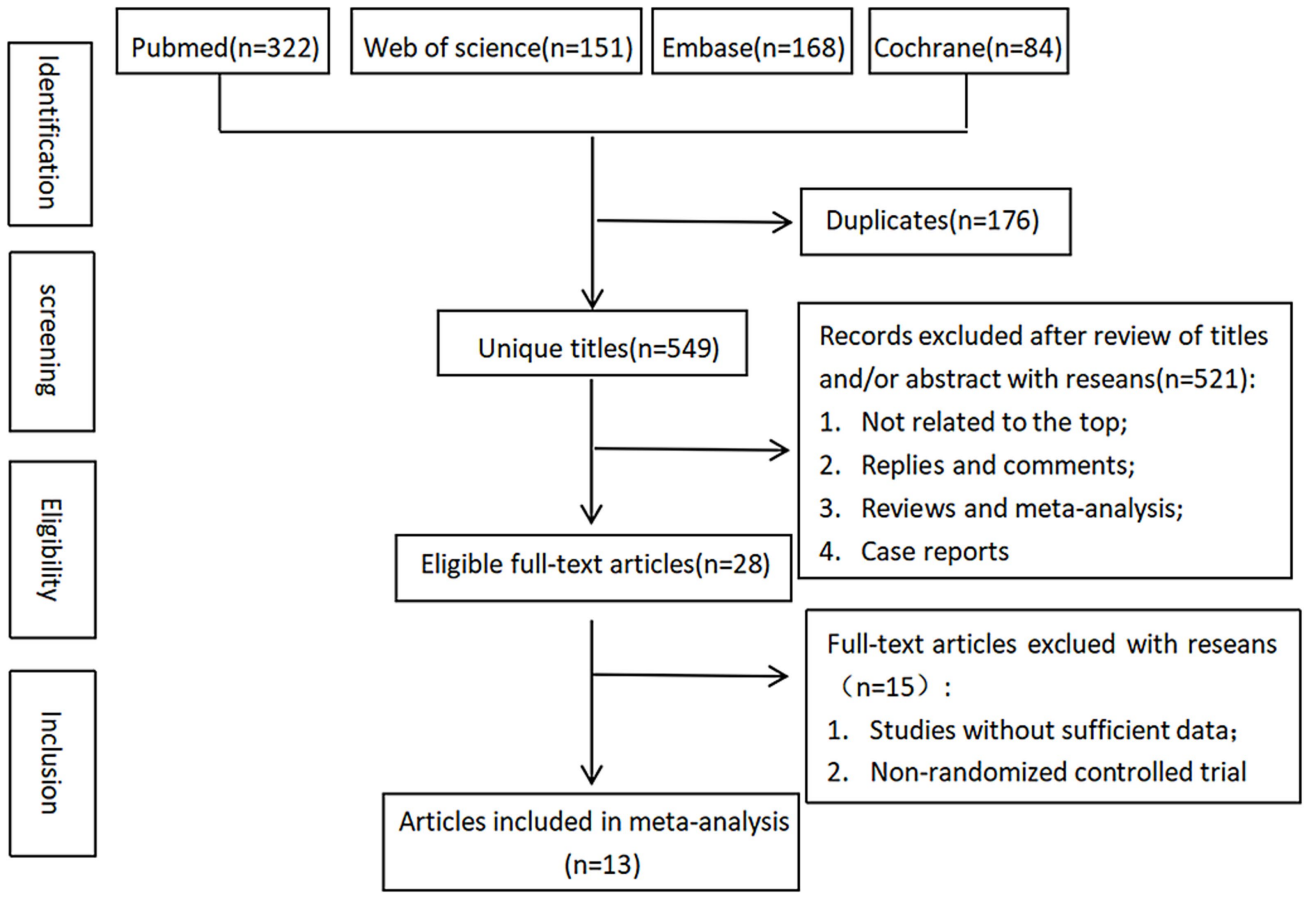
A flowchart of study screening.

A total of 725 relevant studies were retrieved, including 322 from Pubmed, 151 from Web of Science, 168 from Embase, and 84 from Cochrane. Of the retrieved studies, 176 duplicates, 75 reviews, 26 non-primary studies, 6 non-English-language studies, and 66 irrelevant studies were excluded. The remaining 376 studies were initially screened by reading their titles and abstracts, and 348 of them were excluded. 28 studies left were re-screened, and non-RCTs and studies without available data were excluded. In the end, 13 studies (23, 24, 33–43) were included. The flowchart of study screening is shown in Figure 1.

### 3.2 General characteristics of included studies

Thirteen studies in total were included (with 488 subjects: 244 in the control group and 244 in the intervention group). The baseline data of the patients in the included studies, such as age, sex, nationality, type of cognitive impairment, and motor function, as well as other specific details, are provided in Table 1. They used different cognitive assessment tools, including MOCA used in four studies (23, 24, 33, 34), FAB used in five studies (23, 33, 35–37), TMT used in four studies (33, 35, 37, 38), MMSE used in three studies (35, 36, 39), FIM used in three studies (33, 35, 40), RAVLT used in two studies (35, 39), AM used in two studies (23, 39). There were some other evaluation tools, such as HAM-A and HAM-D used in three studies (23, 39, 43), SS-QOL used in two studies (24, 34), and MBI used in two studies (37, 42).

**Table 1:** The literature characteristics were included.

First author and Year	Country	Patients		Mean/median age		Male		Baseline level of cognitive function		Baseline level of motor function		Intervening measure		Intervention time	Outcomes
		Intervention	Control	Intervention	Control	Intervention	Control	Intervention	Control	Intervention	Control	Intervention	Control		
Torrisi (2021) (23)	Italy	24	24	53.2 ± 12.1	55.3 ± 8.7	9 (37.5%)	9 (37.5%)	Moca: 24 (23–27) FAB: 15.0 (13.3–15.8) AM: 42.2 (38–48.5) WEIGL: 8.5 (7–10.2)	Moca:25 (23.7–26) FAB:13.8 (12.9–15) AM:41.8 (33.7–47) WEIGL:9.7 (8.5–11)			Amadeotm hand training	The Cognitive Orientation to daily occupational performance	8 weeks	1.2.3.9
Manuli (2020) (33)	Italy	30	30	48.0 ± 12.1	43.1 ± 9.7	19 (63.3%)	16 (53.3%)	Moca: 21.8 ± 2.7 (RRG + VR); 22.6 ± 2.5 (RRG−VR) FAB:11,1 ± 2.7 (RRG + VR);14.5 ± 2.2 (RRG−VR) TMTA:155.4 ± 84.2 (RRG + VR); 132.3 ± 111.1 (RRG-VR) TMTB:255.3 ± 196.0 (RRG + VR); 226.2 ± 118.0 (RRG-VR)	Moca:23.4 ± 2.4 FAB:14.6 ± 2.0 TMTA:139.3 ± 123.9 TMTB:226.3 ± 128.0	FIM:25.8 ± 2.2(RRG + VR) FIM:26.2 ± 3.5(RRG-VR)	FIM:26.9 ± 4.2	Lokomat training	Training of executive functions was performed by working on categorization, planning, association, and analogical reasoning	8 weeks	1.2.6.7.18
Maier (2017) (35)	Barcelona	6	5	66.33	64.00	4 (66.7%)	2 (40%)	FAB:15.0 (1.64) MMSE:27.5 (2.34) TMT A:79.0 (79.33) TMT B:266.33 (184.83) RAVLTI:27.17 (12.86) RAVLTD:3.83 (3.18)	FAB:15.2 (3.42) MMSE:27.2 (1.64) TMT A:63.6 (39.12) TMT B:265.4 (187.41) RAVLTI:34.80 (11.05) RAVLTD:7.40 (1.94)	FM-UE:48.33 (13.63)	FM-UE:51.00 (27.97)	Vr training	The control group receives a folder with 30 individual cognitive tasks (e.g., draw figures mirrored, complete sentences or word search puzzle etc.).	6 weeks	4.5.6.15
Taravati (2022) (24)	Turkey	22	23	50.94 ± 17.20	55.75 ± 11.61	14 (82.35%)	14 (82.35%)	Moca: 20 (17–24) FIM-Cognitive 19 (13–26)	Moca: 21 (16–22) (FIM)-Cognitive 19.5 (16.5–24)	FIM:92 (63–106)	FIM:86 (58–103)	Reogo™-motorika upper extremity rehabilitation and conventional rehabilitation	The conventional rehabilitation exercises carried out by a physiotherapist consisted of ROM exercises, muscle strengthening, balance and mobility training,	6 weeks	1.7.18
Zengin-Metli (2018) (40)	Turkey	15	20	59.3 ± 8.1	63.3 ± 3.9	15 (75%)	6 (40%)	FIM -Cognitive: 31.85 ± 3.66	FIM -Cognitive: 27.6 ± 7.55	FIM-Motor: 60.75 ± 18	FIM-Motor: 62.67 ± 18.47	Robot-assisted	Conventional program consisted neurophysiological exercises with Brunnstrom approach, range of motion exercises, and postural education.	3 weeks	7.18
Daunoraviciene (2018) (43)	Lithuania	17	17	65.88 ± 4.87	65.47 ± 4.05	11 (64.7%)	11 (64.7%)	ACE-R:73.88 ± 14.32	ACE-R:73.88 ± 14.32	FMA:32.18 ± 16.53	FMA:74.47 ± 9.34	Robot-assisted	Conventional functional rehabilitation for 35–60 min/day in approximately 10 occupational therapy sessions (including, exercising, physical activities, active table games etc.)	5–7 weeks	10.11.14
Zhao (2022) (42)	China	12	13	50.1 ± 11.1	56.1 ± 11.5	13 (92.9%)	12 (85.7)	LOCTA:55.79 (17.81)	LOCTA:57.07 (17.28)	FMA LE:10.43 (5.67)	FMA LE:10.07 (6.83)	Bci-controlled Robot +the international 10–20 system for EEG Recordings and used Newton’s ring as a stimulator	30 min of robot training and 30 min of PT training once a day	4 weeks	8, 13.14
De Luca (2018) (39)	Messina	20	15	43.9 ± 16.6	42.1 ± 17.7	11 (55.0%)	7 (46.7%)	MMSE:22.7 ± 2.5 AM:29.6 ± 15.1 RAVLI: 27.2 ± 13.6 RAVLR 3.9 ± 4.3	MMSE:23.8 ± 3.5 AM:35.3 ± 14.0 RAVLI: 30.1 ± 12.9 RAVLR:4.5 ± 4.9			The pc-based erica training and traditional cognitive rehabilitation	Traditional Cognitive Behavioral Therapy	8 weeks	3.4.5.10.11
Ranzani (2020) (36)	Switzerland	14	13	70.00 ± 12.79	67.46 ± 11.39	10 (71.43%)	8 (61.54%)	MMSE:25.89 (3.60) FAB:14.60 (2.38)	MMSE:23.62 (5.47) FAB:11.98 (5.29)	FMA-UE:50.14 (12.50) FMA-WH:17.86 (5.61) FMA-SE:32.29 (8.08)	FMA-UE:50.85 (15.00) FMA-WH:17.86 (5.61) FMA-SE:31.46 (8.95)	Robot-assisted neurocognitive therapy with the ReHapticKnob haptic device	All subjects received three neurocognitive therapy sessions (2 × 45 min and 1 × 30 min) per day focusing on hand function	32 weeks	2.5.14
Castelli (2023) (37)	Italy	12	12	77.1 ± 11.25	76.6 ± 8.87	7 (58.33%)	7 (58.33%)	FAB:9 (7.75–9.25) TMT:9 (7.75–9.25)	FAB:8 (6–9) TMT:23 (16–29.5)	FAC:2 (2–2.25)	FAC:1.5 (1–3)	Robotic-assisted therapy+ Conventional Rehabilitation	Rehabilitation treatment including strength, balance, gait, and functional training tailored to the individual patient’s needs.	4 weeks	2.6.13.16
Akinci (2024) (34)	Turkey	17	17	59.12 ± 13.22	59.18 ± 8.89	13 (76.5%)	13 (76.5%)	MoCA:16.77 (2.44)	MoCA:16.82 (2.22)			Bothconventional physiotherapy and robot-assisted	Included dynamic stretching, strengthening exercises, mat activities range of motion exercises, static and dynamic balance training, and walking exercises	6 weeks	1
Kim (2020) (38)	Korea	15	15	59.4 ± 1.8	54.73 ± 2.98	7 (46.7%)	7 (46.7%)	TMT:32.87 ± 5.49	TMT:33.20 ± 5.48	JTT:14.73 ± 3.20	JTT:13.13 ± 3.60	Fupper limb sensory Stimulation and virtual reality rehabilitation (smvr)	Conventional conservative treatment and peripheral joint mobilization of the upper limb	8 weeks	6.17

MoCA, montreal cognitive assessment; FAB, frontal assessment battery; AM, attentive matrices; RAVLT, Ray auditory verbal learning test; MMSE, mini-mental-state-examination; TMT, trail making test form; FIM-Cognitive, functional independence measure cognitive; LOTCA, Loewenstein occupational therapy cognitive assessment; WEIGL, Weigl test; HAD-D, the Hamilton rating scale for depression; HAD -A, the Hamilton rating scale for anxiety; SS-QOL, stroke specifc quality of life scale; MBI, Modified Barthel index; FMA, Fugl-Meyer assessment; FM, Fugl-Meyer; FAC, functional ambulation classification; JTT, Jebsen-Taylor hand function test; FIM, functional independence measure.

### 3.3 Assessment of included studies regarding their quality

**Figure 2:**
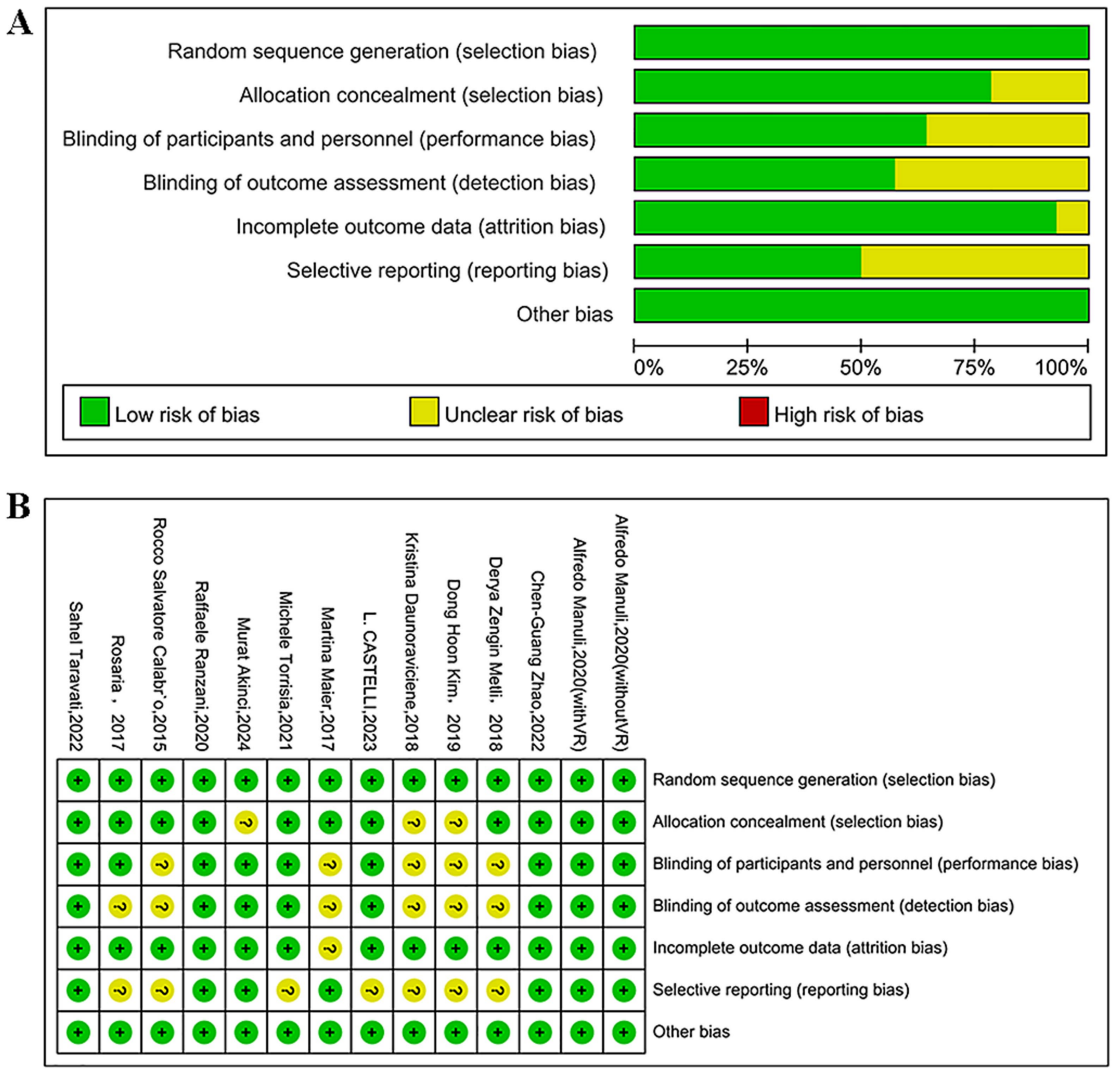
**(A)** Percentages of items at each RoB in the included studies; **(B)** RoB in the included studies.

The quality of the 13 studies (RCTs) included was assessed using the Cochrane RoB tool. The results showed that three studies were assessed as Grade A for quality, and the remaining 10 were all assessed as Grade B, as detailed in Figures 2A,B.

### 3.4 MA results

#### 3.4.1 Changes in primary outcomes

##### 3.4.1.1 Change in MoCA score

Four studies (23, 24, 33, 34) assessed the effect of RAT on MOCA, including a total of 247 patients. Statistical heterogeneity among them was observed (*p* = 0.06, *I*^2^ = 56%). MA using the REM showed a significant improvement in post-intervention MoCA score in the test group compared to the control group, with a statistical difference [SMD = 0.43, 95% CI (0.04–0.81), *p* = 0.03], as shown in Supplementary Figure S1.

##### 3.4.1.2 Change in FAB score

Five studies (23, 33, 35–37) assessed the effect of RAT on FAB, including a total of 230 patients. Statistical heterogeneity among them was observed (*p* = 0.001, I^2^ = 75%). MA using the REM showed no statistically significant intergroup difference in the post-intervention FAB score [SMD = 0.47, 95% CI (−0.09–1.04), *p* = 0.10], as shown in Supplementary Figure S2.

##### 3.4.1.3 Change in MMSE score

Three studies (35, 36, 39) assessed the effect of RAT on MMSE, including a total of 73 patients. No statistical heterogeneity among them was observed (*p* = 0.41, *I*^2^ = 0%). MA using the REM showed no statistically significant intergroup difference in the post-intervention MMSE score [SMD = 0.25, 95% CI (−0.22 to 0.17), *p* = 0.30] (Supplementary Figure S3).

##### 3.4.1.4 Change in AM score

Two studies (23, 39) examined the effect of RAT on AM, encompassing 84 patients. No statistical heterogeneity among them was observed (*p* = 0.45, *I*^2^ = 0%). MA using the REM showed no statistically significant intergroup difference in the post-intervention AM score [SMD = −0.39, 95% CI (−0.05 to 0.82), *p* = 0.08] (Supplementary Figure S4).

##### 3.4.1.5 Change in RAVLT-immediate score

Two studies (35, 39) assessed the effect of RAT on RAVLT-immediate score, including a total of 46 patients. No statistical heterogeneity among them was observed (*p* = 0.85, *I*^2^ = 0%). MA using the REM showed no statistically significant intergroup difference in the post-intervention RAVLT-immediate score [SMD = −0.12, 95% CI (−0.47 to 0.70), *p* = 0.70], as shown in Supplementary Figure S5.

##### 3.4.1.6 Change in RAVLT delayed recall score

Two studies (35, 39) reported RAVLT Delayed Recall, with a total of 46 patients. No statistical heterogeneity among them was observed (*p* = 0.85, *I*^2^ = 0%). MA using the REM showed no statistically significant intergroup difference in the post-intervention RAVLT Delayed Recall score [SMD = −0.02, 95% CI (−0.60 to 0.57), *p* = 0.96] (Supplementary Figure S6).

##### 3.4.1.7 Change in TMT score

The four studies (33, 35, 37, 38) examined the effect of RAT on TMT, with a total of 200 patients. No statistical heterogeneity among them was observed (*p* = 0.56, *I*^2^ = 0%). MA using the REM showed no statistically significant intergroup difference in the post-intervention TMT score [SMD = 0.18, 95% CI (−0.47 to 0.11), *p* = 0.22] (Supplementary Figure S7).

#### 3.4.2 Changes in secondary outcomes

##### 3.4.2.1 Change in FIM score

Three studies (24, 33, 40) assessed the effect of RAT on the FIM, including a total of 200 patients. No statistical heterogeneity among them was observed (*p* = 0.001, *I*^2^ = 81%). MA using the REM showed no statistically significant intergroup difference in the post-intervention FIM score [SMD = 0.57, 95% CI (−0.10 to 0.25), *p* = 0.10] (Supplementary Figure S8).

##### 3.4.2.2 Change in HAM-A score

Three studies (23, 39, 43) assessed the effect of RAT on HAM-A, and they included a total of 200 patients. Statistical heterogeneity among them was observed (*p* < 0.00001, *I*^2^ = 93%). MA using the REM showed no statistically significant intergroup difference in the post-intervention HAM-A score [SMD = −0.97, 95% CI (−2.49 to 0.56), *p* = 0.22] (Supplementary Figure S9).

##### 3.4.2.3 Change in HAM-D score

Three studies (23, 39, 43) investigated the effect of RAT on HAM-D, including a total of 200 patients. Statistical heterogeneity among them was observed (*p* < 0.00001, *I*^2^ = 93%). MA using the REM showed no statistically significant intergroup difference in the post-intervention HAM-D score [SMD = −0.94, 95% CI (−2.48 to 0.60), *p* = 0.23] (Supplementary Figure S10).

##### 3.4.2.4 Change in SS-QOL score

Two studies (24, 34) reported SS-QOL, including a total of 80 patients. No statistical heterogeneity among them was observed (*p* = 0.75, *I*^2^ = 0%). MA using the REM showed no statistically significant intergroup difference in the post-intervention SS-QOL score [SMD = −0.23, 95% CI (−0.21 to 0.67), *p* = 0.30] (Supplementary Figure S11).

##### 3.4.2.5 Change in MBI score

Two studies (37, 42) focused on MBI, with a total of 80 patients. No statistical heterogeneity among them was observed (*p* = 0.05, *I*^2^ = 74%). MA using the REM showed no statistically significant intergroup difference in the post-intervention MBI score [SMD = −0.88, 95% CI (−0.31 to 2.06), *p* = 0.15] (Supplementary Figure S12).

### 3.5 Sensitivity analysis

Sensitivity analyses were executed for the change in MoCA, FAB, MMSE, TMT, HAM-A, or HAM-D score by the one-by-one elimination method. The results revealed significantly decreased heterogeneity (*p* = 0.35, *I*^2^ = 4%) for the change in MoCA score after the exclusion of one study (33) using RAT plus VR training, suggesting that this study was the main source of the heterogeneity for the change in MoCA score. In addition, after the exclusion of this study, the inter-group difference in the change in MoCA score changed from being significant to being not significant, suggesting unstable results of change in MoCA score. After the exclusion of one study (33) using RAT without VR, the inter-group difference in the change in FAB score changed from being not significant to being significant [SMD = 0.64, 95% CI (0.09–1.2), *p* = 0.02], suggesting unstable results of change in FAB score. After the exclusion of one study (33) using RAT with VR, significantly decreased heterogeneity (*p* = 0.18, *I*^2^ = 41%) was observed for the change in FIM score, suggesting that this study was the main source of heterogeneity for the change in FIM score. Regarding the changes in other scores, there was no difference in each combined effect size after the exclusion of each single study, indicating basically robust and reliable MA results. The results of the SEN analysis are presented in Supplementary Figure S13.

### 3.6 Publication bias analysis

The funnel plots were symmetric, suggesting no publication bias for the changes in MoCA, FAB, FIM, MMSE, TMT, HAM-A, and HAM-D scores, as shown in Supplementary Figure S14. The Egger’s test results showed no publication bias for the changes in MoCA (*p* = 0.956), FAB (*p* = 0.749), FIM (*p* = 0.613), TMT (*p* = 0.578), MMSE (*p* = 0.270), HAM-A (*p* = 0.089), and HAM-D (*p* = 0.253) scores in the included studies.

### 3.7 Subgroup analysis

Changes in FAB and MoCA scores were subjected to subgroup analysis by age, intervention, and duration of intervention. Subgroup analysis by age or duration of intervention showed no statistically significant differences in the change in post-intervention FAB score. However, subgroup analysis by intervention showed that robot-assisted +VR training significantly improved FAB score in patients. Subgroup analysis by the duration of intervention or intervention showed no statistically significant differences in the change in post-intervention MoCA score. The results of the subgroup analysis are detailed in Table 2.

**Table 2:** Subgroup analysis.

Subgroup	Weigl test and frontal Assessment Battery (FAB)				Montreal cognitive Assessment (MoCA)			
	Study	SMD [95% CI]	*p* value	*I* ^2^	Study	SMD [95% CI]	*p* value	*I* ^2^
Total	6	0.47 [−0.09–1.04]	0.1	75%	5	0.43 [0.04–0.81]	0.03	56%
Mean/median age								
≥60 y	3	0.69 [−0.26–1.63]	0.16	89%	0			
<60 y	3	0.24 [−0.26–0.75]	0.33	0%	5			
Follow-up								
≥8 weeks	4	0.56 [−0.2–1.31]	0.15	84%	3	0.41 [−0.18–1.0]	0.17	72%
<8 weeks	2	0.33 [−0.32–0.98]	0.32	0%	2	0.46 [−0.13–1.05]	0.13	42%
Intervention								
Robot-assisted	3	0.08 [−0.30–0.46]	0.68	2%	2	0.35 [−0.38–1.08]	0.34	71%
Robot-assisted + VR	3	0.82 [0.02–1.61]	0.04	72%	3	0.48 [−0.06–1.02]	0.08	60%

### 3.8 GRADE assessment

Based on the GRADE results, the quality of the evidence of TMT, MMSE, AM, RAVLT-immediate, RAVLT-Delayed Recal, and SS-QOL indicators were moderate, while the quality of the evidence of MOCA, FAB, FIM, HAD-AHAD-D, and MBI indicators were low. Detailed GRADE results are shown in Table 3.

**Table 3:** GRADE rating of each outcome.

No. of studies	Outcomes	SMD	95% CI	*I*^2^; *p* value	Risk of bias	Inconsistency	Indirectness	Imprecision	Publication bias	Plausible confounding	Magnitude of effect	Dose–response gradient	Grade
4	Montreal Cognitive Assessment (MoCA)	0.43	0.04, 0.81	56%; *p* = 0.06	No serious risk	Serious inconsistency	No serious indirectness	Serious imprecision	Undetected	Would not reduce effect	No	No	Low
5	Weigl test and Frontal Assessment Battery (FAB)	0.64	0.19, 1.20	75%; *p* = 0.001	No serious risk	Serious inconsistency	No serious indirectness	Serious imprecision	Undetected	Would not reduce effect	No	No	Low
4	Trail Making Test Form (TMT)	−0.18	−0.47, 0.11	0%; *p* = 0.56	No serious risk	No serious inconsistency	No serious indirectness	Serious imprecision	Undetected	Would not reduce effect	No	No	Moderate
3	Functional Independence Measure (FIM)	0.57	−0.1, 0.25	81%; *p* = 0.001	No serious risk	Serious inconsistency	No serious indirectness	Serious imprecision	Undetected	Would not reduce effect	No	No	Low
3	Mini-Mental-State-Examination (MMSE)	0.25	−0.22, 0.71	0%; *p* = 0.41	No serious risk	No serious inconsistency	No serious indirectness	Serious imprecision	Undetected	Would not reduce effect	No	No	Moderate
3	Hamilton Rating Scale for Anxiety (HAD-A)	−0.97	−2.49, 0.56	93%; *p* < 0.00001	No serious risk	Serious inconsistency	No serious indirectness	Serious imprecision	Undetected	Would not reduce effect	No	No	Low
3	Hamilton Rating Scale for Depression (HAD-D)	−0.94	−2.48, 0.60	93%; *p* < 0.00001	No serious risk	Serious inconsistency	No serious indirectness	Serious imprecision	Undetected	Would not reduce effect	No	No	Low
2	Attentive Matrices (AM)	−0.39	−0.05, 0.82	0%; *p* = 0.45	No serious risk	No serious inconsistency	No serious indirectness	Serious imprecision	NA	Would not reduce effect	No	No	Moderate
2	Ray Auditory Verbal Learning Test (RAVLT)-immediate	0.12	−0.47, 0.70	0%; *p* = 0.85	No serious risk	No serious inconsistency	No serious indirectness	Serious imprecision	NA	Would not reduce effect	No	No	Moderate
2	Ray Auditory Verbal Learning Test (RAVLT)- Delayed Recal	−0.02	−0.60, 0.57	0%; *p* = 0.85	No serious risk	No serious inconsistency	No serious indirectness	Serious imprecision	NA	Would not reduce effect	No	No	Moderate
2	Stroke Specifc Quality of Life Scale (SS-QOL)	0.23	−0.21, 0.67	0%; *p* = 0.75	No serious risk	No serious inconsistency	No serious indirectness	Serious imprecision	NA	Would not reduce effect	No	No	Moderate
2	Modified Barthel Index (MBI)	0.88	−0.31, 2.06	74%; *p* = 0.05	No serious risk	Serious inconsistency	No serious indirectness	Serious imprecision	NA	Would not reduce effect	No	No	Low

## 4 Discussion

About 40% of stroke patients may develop mild cognitive impairments in the first year after stroke. The incidence of cognitive impairment was 10% after the first stroke and increased to 33% after a recurrent stroke. Compared with other neurological deficits including sensory or motor impairment, cognitive impairment after stroke is a frequent but neglected consequence. It is, therefore, crucial to assess not only physical and motor skill impairments, but also QoL and CF (41). As the number of stroke patients increases, it can create an imbalance in the healthcare system (largely because of a shortage of caregivers or physical therapists), and robots can be a possible solution. Evidence suggests that rehabilitation robots are at least as effective as traditional training (42). Usually, functional motor recovery in upper limbs after stroke is achieved within the first 6 months after upper-limb functional decline in any chronic phase after stroke (43–45). The maximum improvement in overall CF, particularly attention, memory, and visuospatial ability, is observable in the first 4 months of PS (11). Cognitive impairment seriously affects a person’s ADL (46). It is therefore important to choose the most appropriate and effective strategy for rehabilitation in the acute PS period.

Previously, more focus had been put on the effect of RAT on motor function and QoL in PS patients, whereas there were fewer studies on the effect of RAT on CF in PS patients. Currently, no MA or review of the effect of RAT on CF after stroke has been published. All subjects in the 13 studies (RCTs) included in this study completed trials, without adverse events. This study showed that a statistically significant difference was only observed in the MoCA score in PS patients with RAT, possibly because of the small number of included studies. SEN analysis showed that after the exclusion of one study (33) using RAT combined with VR, significantly decreased heterogeneity (*p* = 0.35, *I*^2^ = 4%) was observed for the change in MoCA score, suggesting that this study was the main source of heterogeneity for this indicator. In addition, after the exclusion of this study, the inter-group difference in the change in MoCA score changed from being significant to being not significant, suggesting unstable results of this indicator. After excluding one study (33) using RAT without VR, the inter-group difference in the change in FAB score changed from being not significant to being significant [SMD = 0.64, 95% CI (0.09–1.2), *p* = 0.02], suggesting an unstable result of the change in FAB score. After excluding one study (33) using RAT combined with VR, significantly decreased heterogeneity (*p* = 0.18, *I*^2^ = 41%) was observed for the change in FIM score, suggesting that this study was the main source of heterogeneity for this indicator. Regarding the changes in other scores, there were no statistical differences in the combined effect size after excluding each single study, indicating basically robust and reliable results of MA.

Regarding most cognitive assessment scores, there was an improvement in outcomes in the test group compared to the control group. Of the studies included in this analysis, five used RAT plus VR training as an intervention, seven used RAT alone, and one used computerized training. Of the included studies, the study by Taravati et al. (24) showed that compared with the control group, robotic rehabilitation improved function scores, spasticity, general functioning, ADL, and cognitive functions in PS patients, but this difference was not statistically significant. In addition, the study by Torrisi (23) suggested that both motor function in the paretic arm and global/specific cognitive abilities in the chronic phase in PS patients can be enhanced by RAT and VR-based hand function rehabilitation. Similarly, the study by Manuli et al. (33) showed that compared with RAT alone or traditional training, VR-based RAT significantly improved CF after stroke. The findings of the above studies are not entirely consistent with the conclusion of this study. The results of this study reveal that the difference between RAT and traditional training is not significant in terms of improving CF. This difference possibly arose from high heterogeneity among the included studies, their small number/small sample size, and a short study period possibly insufficient to demonstrate the true efficacy of RAT in the PS population.

RAT provides patients with intensive, repetitive, task-oriented motor exercises, which require research, time, and planning skills of patients (47, 48). RAT is sufficient to powerfully stimulate the mechanisms of neuroplasticity, which are central to the recovery of motor function and CF after brain injury, especially in a chronic phase (39, 49). In addition, providing VR-based feedback to patients may contribute to better outcomes, due to more sensorimotor information and cognitive processes in which patients are more involved (50–55). VR is able to achieve the maximum therapeutic effect by performing exercises in VR, as action observation can activate the human mirror neuron system, enhance motor learning, and induce profound cortical and subcortical changes at cellular and synaptic levels. Thus, multisensory feedback and repetitive implementation via robots combined with VR can improve patient prognosis and enhance both motor function and CF. Sensory-motor-cognitive processes depend on overlapping neuronal circuits, which involve the thalamus and basal ganglia and then are connected to the brainstem and cerebellum, the latter of which plays a pivotal role in cognition, beyond motor control and balance (56). In improving motor function, QoL, cognition, and emotional state, robotic rehabilitation is similar to traditional rehabilitation of upper limbs in PS patients. Robotic rehabilitation offers a favorable alternative with minor benefits and advantages in work capacity and psychological recovery. Consequently, in addition to traditional neurorehabilitation, it is effective and useful in the management of patients after stroke or cerebrovascular events.

This study still has some limitations. First, its sample size is small. A large number of high-quality studies are still needed for validation. Second, the rehabilitation process is often long, whereas the duration of training is relatively short and insufficient to demonstrate the true efficacy of RAT in improving CF in the PS population. At the same time, no long-term follow-up is conducted. Third, most of the included study populations are from Europe. It is not yet known whether RAT is effective for Asian and African patients, which needs to be further confirmed. Currently, there is still a lot of room for the development of robot-assisted rehabilitation therapy to ameliorate CF in PS patients, such as robot-assisted therapy in combination with VR technology, electromechanical technology, or biofeedback technology. With the development of science and technology, the development of robotic functions will be continuously upgraded and improved to provide patients with smarter and more comprehensive rehabilitation treatment, so as to promote the recovery of various functions in PS patients. This study provides the best evidence regarding the role of RAT in post-stroke cognitive rehabilitation by systematically integrating existing RCTs. Although the meta-analysis showed a trend of improvement in some cognitive indicators (such as MoCA), these findings should be interpreted with caution. Given high heterogeneity, small sample size, and limitations of short-term interventions, the current quality of evidence remains moderate to low, and the stability of results is significantly affected by individual studies. This study provides evidence of this emerging field, rather than drawing definitive conclusions. It reveals the impact of intervention modalities (such as the potential enhanced effects of RAT combined with VR) and heterogeneity in populations. Furthermore, existing research regarding long-term follow-up and standardized assessment is limited. Future research should focus on designing more rigorous multicenter trials, extending the intervention period, and exploring the personalized application of RAT in specific stroke subgroups, aiming to provide more reliable evidence for clinical practice.

## 5 Conclusion

This study reveals that compared with traditional training; RAT has potential value in improving cognitive function in patients with PS. RAT is a safe and effective rehabilitation approach, which enhances not only motor function but also cognitive ability in PS patients. A series of high-quality, large-scale, multicenter RCTs are needed to explore the use of RAT to enhance cognitive recovery in PS patients, so as to obtain broadly representative data and provide robust, reliable evidence for clinical decision-making. Based on such studies, we hope to more accurately evaluate the efficacy and safety of RAT for cognitive rehabilitation of PS patients, thereby promoting the optimization and popularization of treatment regimens.

## Data Availability

The original contributions presented in the study are included in the article/Supplementary material, further inquiries can be directed to the corresponding author.
