# Supplementary material for: Effect of robot-assisted training on cognitive function in post-stroke patients: a meta-analysis
Source: Front Neurol. 2026 Mar 3;17:1725457. doi: 10.3389/fneur.2026.1725457 (PMC12992045; doi:10.3389/fneur.2026.1725457)
Supplement: Supplementary file 1 [file Table_1.docx]

**File S1.** Search strategy

robot-assisted

Robot

Robotic

cognitive function

Stroke

Cognitions

Cognitive Function

Cognitive Functions

Function, Cognitive

Functions, Cognitive

Strokes

Cerebrovascular Accident

Cerebrovascular Accidents

CVA

CVAs

Cerebrovascular Apoplexy

Brain Vascular Accident

Brain Vascular Accidents

Cerebrovascular Stroke

Cerebrovascular Strokes

Apoplexy

Cerebral Stroke

Cerebral Strokes

Acute Stroke

Acute Strokes

Acute Cerebrovascular Accident

Acute Cerebrovascular Accidents

Pubmed-322

**(((Robot) OR (Robotic)) AND (("Cognition"[Mesh]) OR (((((Cognitions) OR (Cognitive Function)) OR (Cognitive Functions)) OR (Function, Cognitive)) OR (Functions, Cognitive)))) AND (("Stroke"[Mesh]) OR (((((((((((((((((Strokes) OR (Cerebrovascular Accident)) OR (Cerebrovascular Accidents)) OR (CVA)) OR (CVAs)) OR (Cerebrovascular Apoplexy)) OR (Brain Vascular Accident)) OR (Brain Vascular Accidents)) OR (Cerebrovascular Stroke)) OR (Cerebrovascular Strokes)) OR (Apoplexy)) OR (Cerebral Stroke)) OR (Cerebral Strokes)) OR (Acute Stroke)) OR (Acute Strokes)) OR (Acute Cerebrovascular Accident)) OR (Acute Cerebrovascular Accidents)))**

Web of science-151

**(((Robot) OR (Robotic)) AND ((Cognition) OR (((((Cognitions) OR (Cognitive Function)) OR (Cognitive Functions)) OR (Function, Cognitive)) OR (Functions, Cognitive)))) AND ((Stroke) OR (((((((((((((((((Strokes) OR (Cerebrovascular Accident)) OR (Cerebrovascular Accidents)) OR (CVA)) OR (CVAs)) OR (Cerebrovascular Apoplexy)) OR (Brain Vascular Accident)) OR (Brain Vascular Accidents)) OR (Cerebrovascular Stroke)) OR (Cerebrovascular Strokes)) OR (Apoplexy)) OR (Cerebral Stroke)) OR (Cerebral Strokes)) OR (Acute Stroke)) OR (Acute Strokes)) OR (Acute Cerebrovascular Accident)) OR (Acute Cerebrovascular Accidents))) (Topic)**

**Embase-168**

**
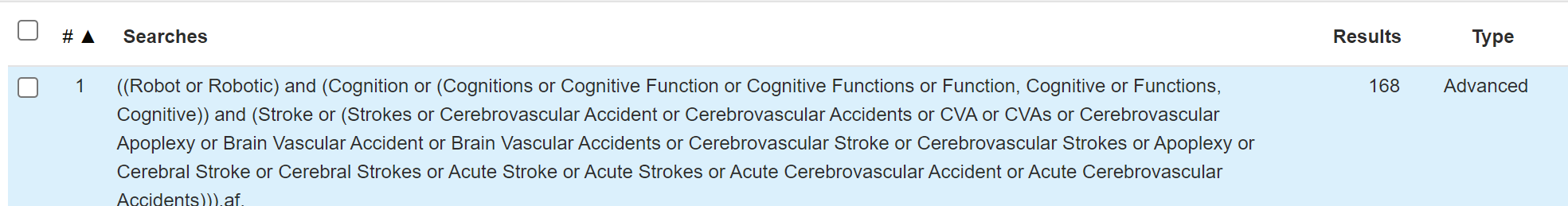
**

**Cochrane-84**

**
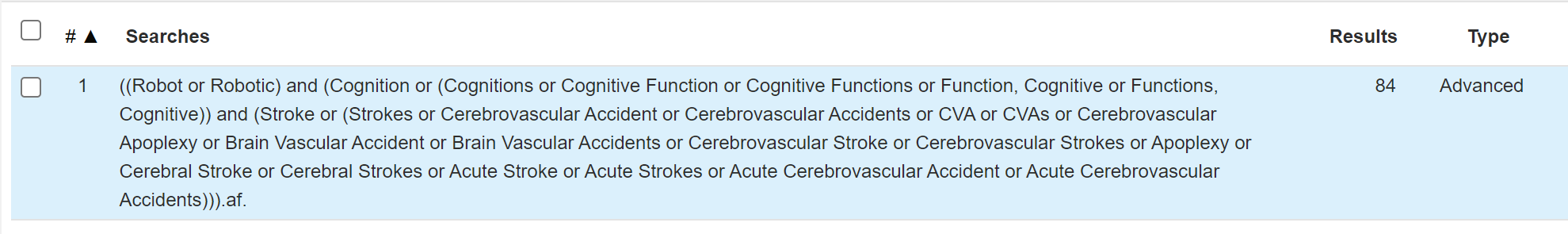
**

**File S2** Characteristics of robot-assisted rehabilitation training programs

| **First author** | **Robot equipment model** | **Intervention duration** | **Intervention frequency** | **Duration of session** | **The key elements of robot-assisted training** |
| --- | --- | --- | --- | --- | --- |
| Michele | Amadeo™ | 8weeks | Five times a week | 1 hour each session | The patient is committed to perform practical, attentive, and visual spatial tasks in a semi-virtual setting |
| Alfredo | Lokomat | 8weeks | Five times a week | 1 hour each session | RRG+VR and RRG-VR were submitted to rehabilitation with the Lokomat device, respectively the Pro and Nanos type |
| Martina | the RGS set-up | 6 weeks | Five times a week | 30 minutes each session | The experimental training consisted of three cognitive training scenarios with a duration of 10 minutes each using the RGS set-up that was stationary in the hospital |
| Sahel | Reogo™-Motorika | 6 weeks | Five times a week | 30-45 minutes each session | The system features 2D and 3D movement selection features. Continuous passive movement, Active-assisted movement, and Active-resistant movement are the most common movement types of the device |
| Derya | Armeo Spring HocomAG Inc. | 3 weeks | Five times a week | 30 minutes each session | Assistive component of the robot was set as tailor as to the subject’s clinical status. The program (game, duration, level of difficulty) was individualized according to the patient’s ability and motor stage. |
| Kristina | The Armeo Spring | 5-7weeks | Five times a week | 30 minutes each session | The Armeo Spring is an instrumented arm orthosis with five degrees of freedom (without robotic actuators) that allows passive movements The instrument contains a spring mechanism for adjustable arm weight support in a large 3D workspace that can be used as a real-time input device to the associated therapy software (Armeo control). |
| Zhao | The BCI-controlled robot | 4 weeks | Six times a week | 30minutes each session | In level 1, the patients could start a training game with the BCI system. In level 2, they manipulated running with left/right turn or accelerating, while, in level 3, they could compete running with obstacles. Meanwhile we adopted the international 10–20 system for EEG recordings, which included O1, O2, Oz, PO3, PO4, and POz sites |
| Rosaria | An Italian computerized cognitive tool, Erica | 8 weeks | Six times a week | 45 minutes each session | Pc-cognitive training was realized by means of an Italian computerized cognitive tool, Erica which consists of a number of personalized pc exercises, articulated in 5 specific cognitive domains |
| Raffaele | ReHapticKnob | 4 weeks (15 times) | 3-4 times a week | 45 minutes each session | Focusing on haptic and postural perception, often without vision, subjects are asked to explore objects (e.g. sponges, sticks, springs), discriminate their properties and perceive relative differences. A robotic device is an ideal tool to perform such exercises, as a wide range of haptic stimuli can easily and accurately be rendered in a repeatable and well-controlled manner |
| L. CASTELLI | Hunova robotic platform | 4 weeks | Three times a week | Unclear | Static and dynamic balance training, trunk control, lower limb function, dual-task training, personalized adjustment. |
| Murat | Lokomat(Hocoma AG) | 6 weeks | Three times a week | 30minutes each session | VR-integrated gait training with 9 interactive tasks; dynamic adjustment of Guidance Force, Body Weight Support, and Walking Speed. |
| Kim | RAPAEL Smart Glove™ (Neofect) | 8 weeks | Three times a week | 60minutes each session | 30 minutes of sensorimotor stimulation (including tactile stimulation, hand therapy, sensory treatment, and functional activities); 30 minutes of VR training (using a smart glove for gamified, task-oriented upper limb motor training). |

**
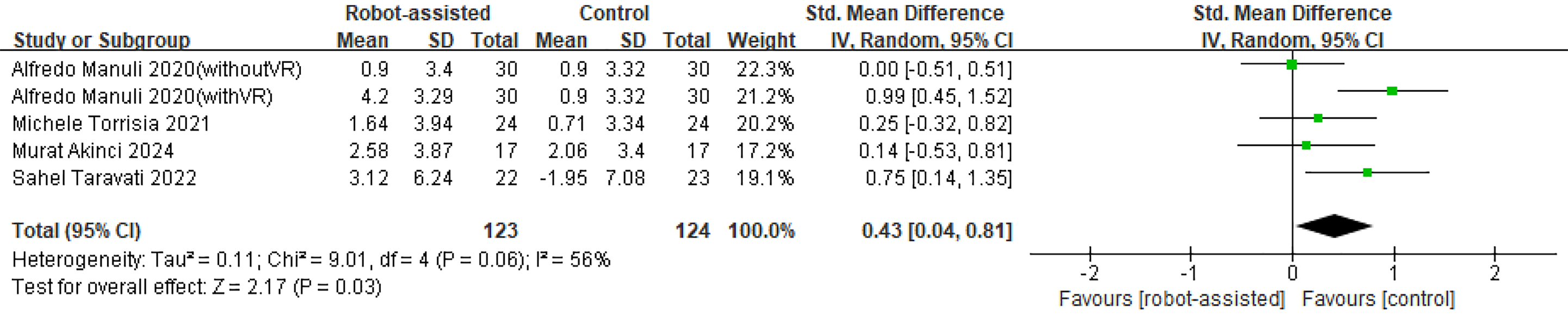
**

**Figure S1** Forest plot for the post-intervention MoCA score in the test group vs control group.


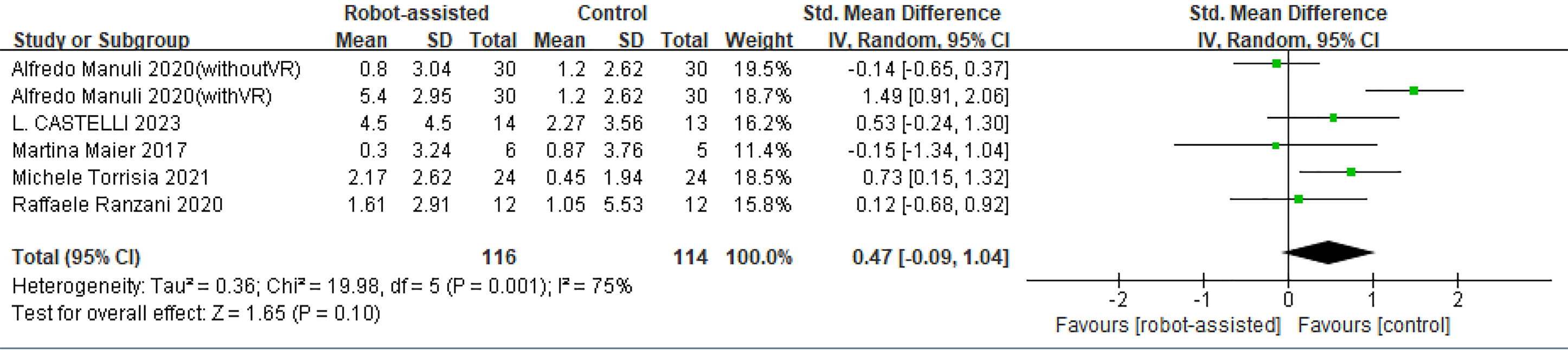


**Figure S2** Forest plot for the post-intervention FAB score in the test group vs control group.


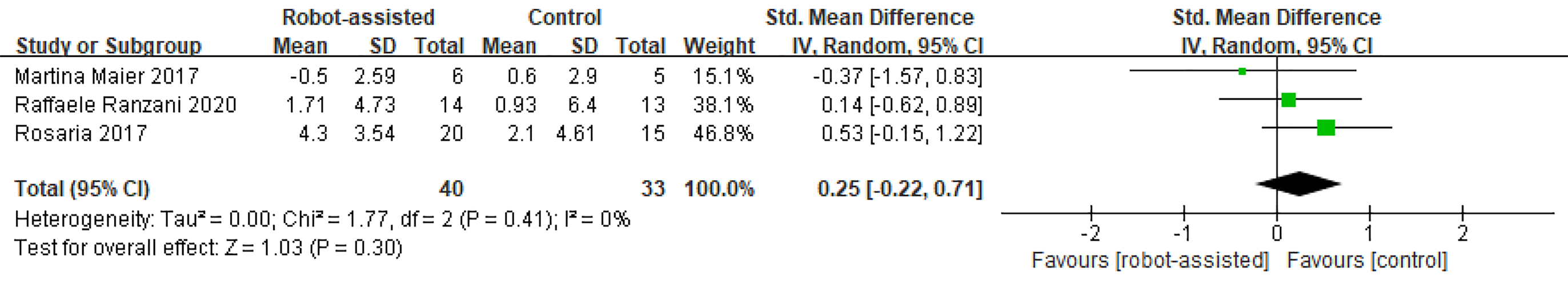


**Figure S3** Forest plot for the post-intervention MMSE score in the test group vs control group.


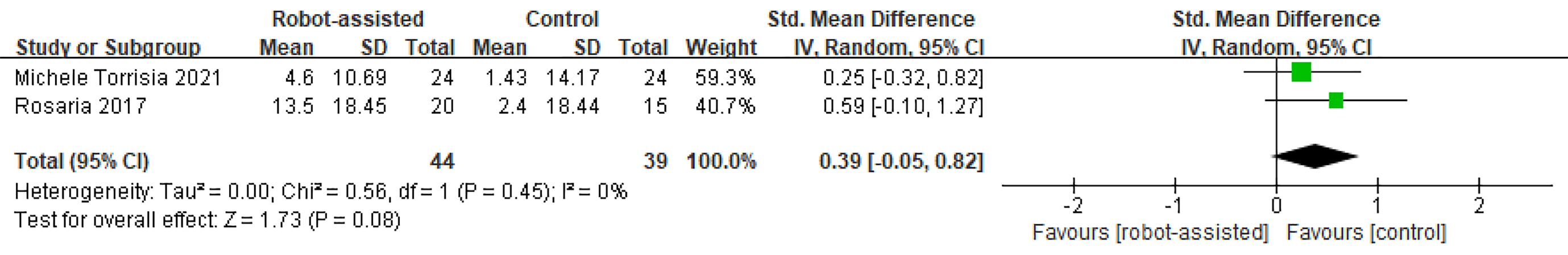


**Figure S4** Forest plot for the post-intervention AM score in the test group vs control group.


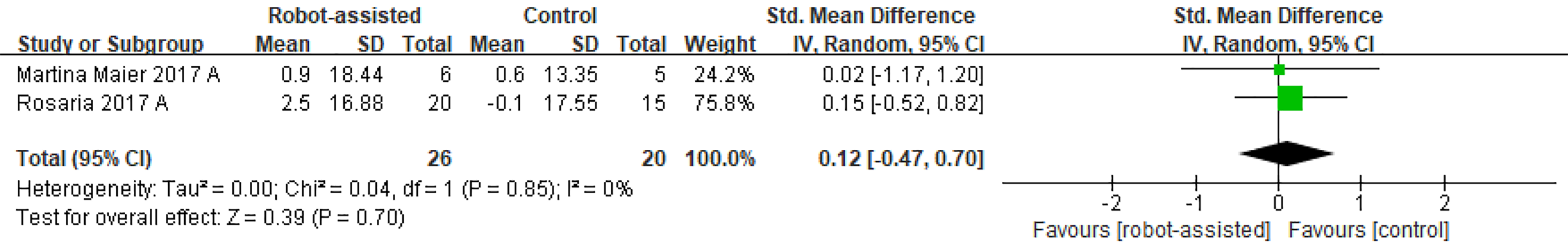


**Figure S5** Forest plot for the post-intervention RAVLT-immediate score in the test group vs control group.


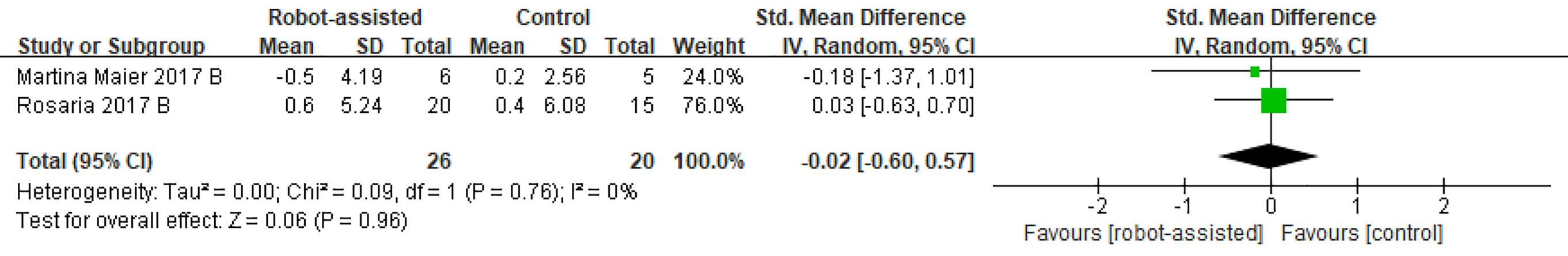


**Figure S6** Forest plot for the post-intervention RAVLT Delayed Recall score in the test group vs control group.


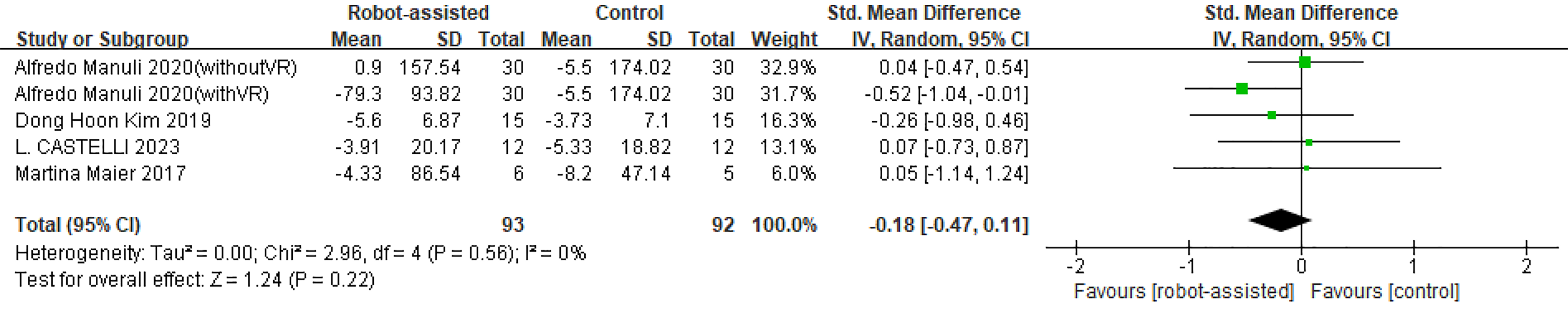


**Figure S7** Forest plot for the post-intervention TMT score in the test group vs control group.


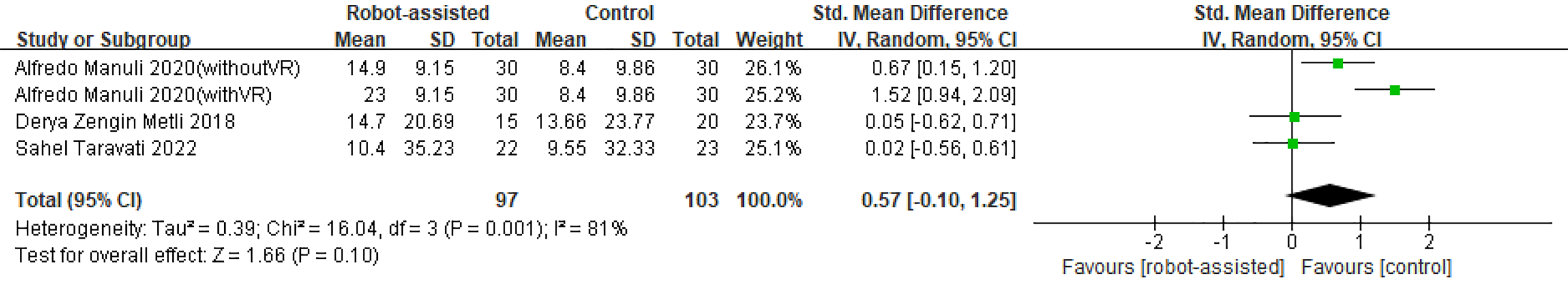


**Figure S8** Forest plot for the post-intervention FIM score in the test group vs control group.


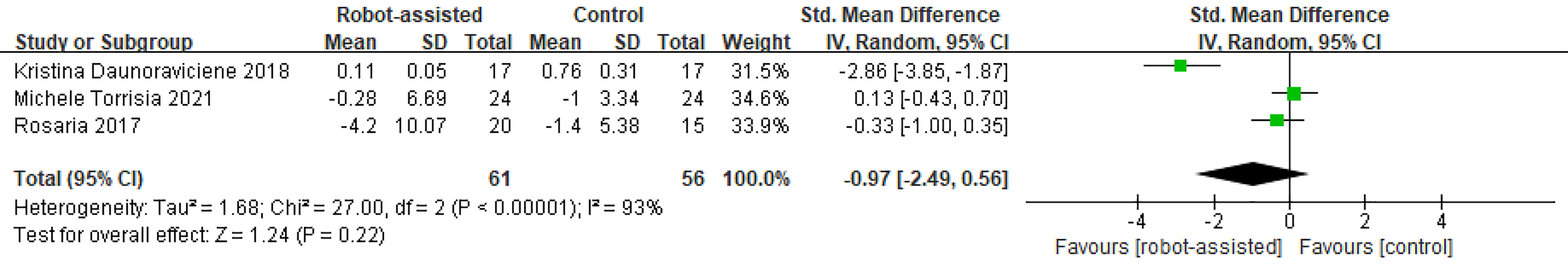


**Figure S9** Forest plot for the post-intervention HAM-A score in the test group vs control group.


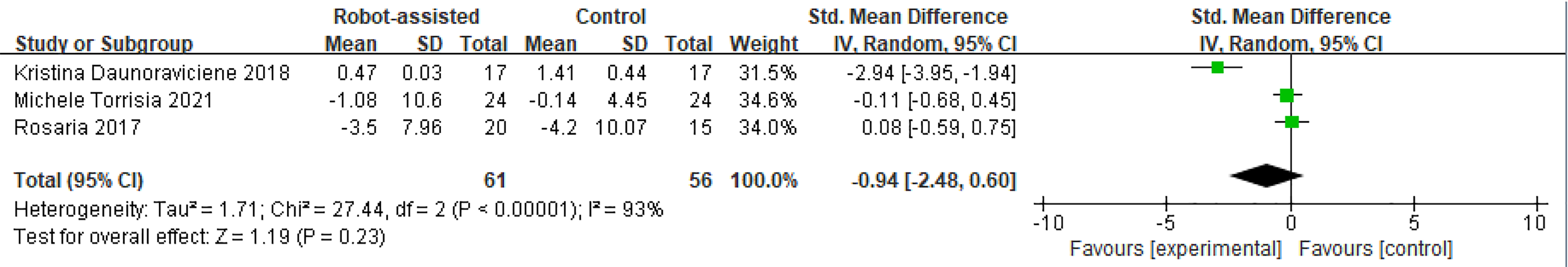


**Figure S10** Forest plot for the post-intervention HAM-D score in the test group vs control group.


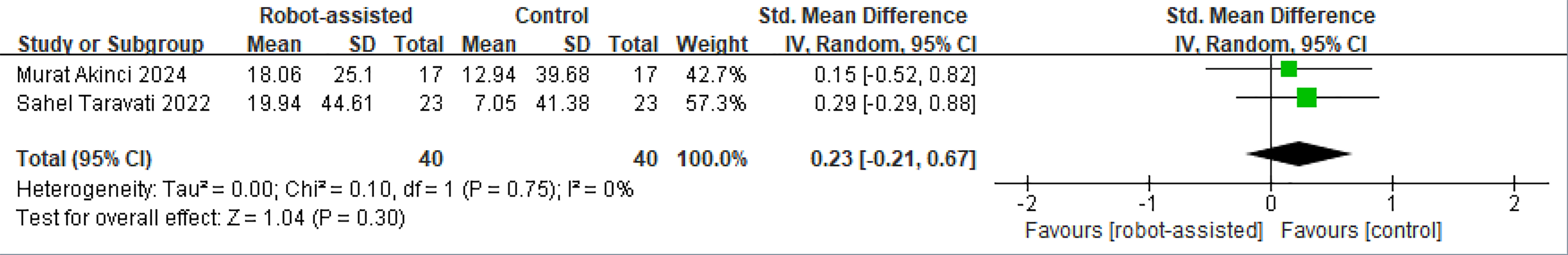


**Figure S11** Forest plot for the post-intervention SS-QOL score in the test group vs control group.


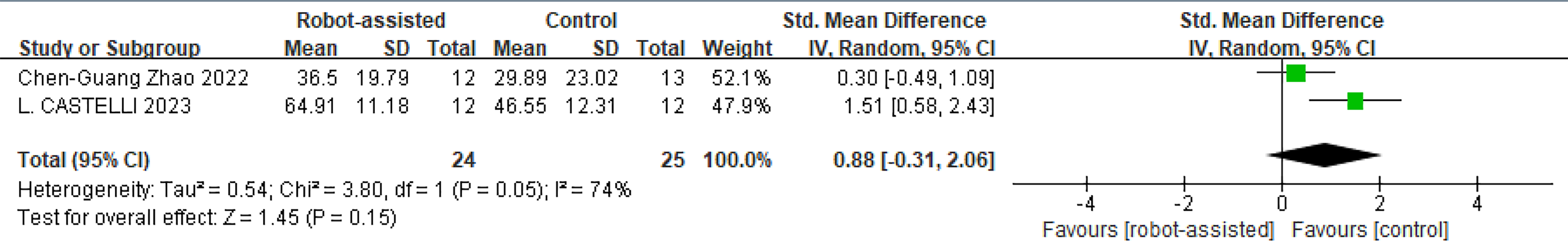


**Figure S12** Forest plot for the post-intervention MBI score in the test group vs control group.


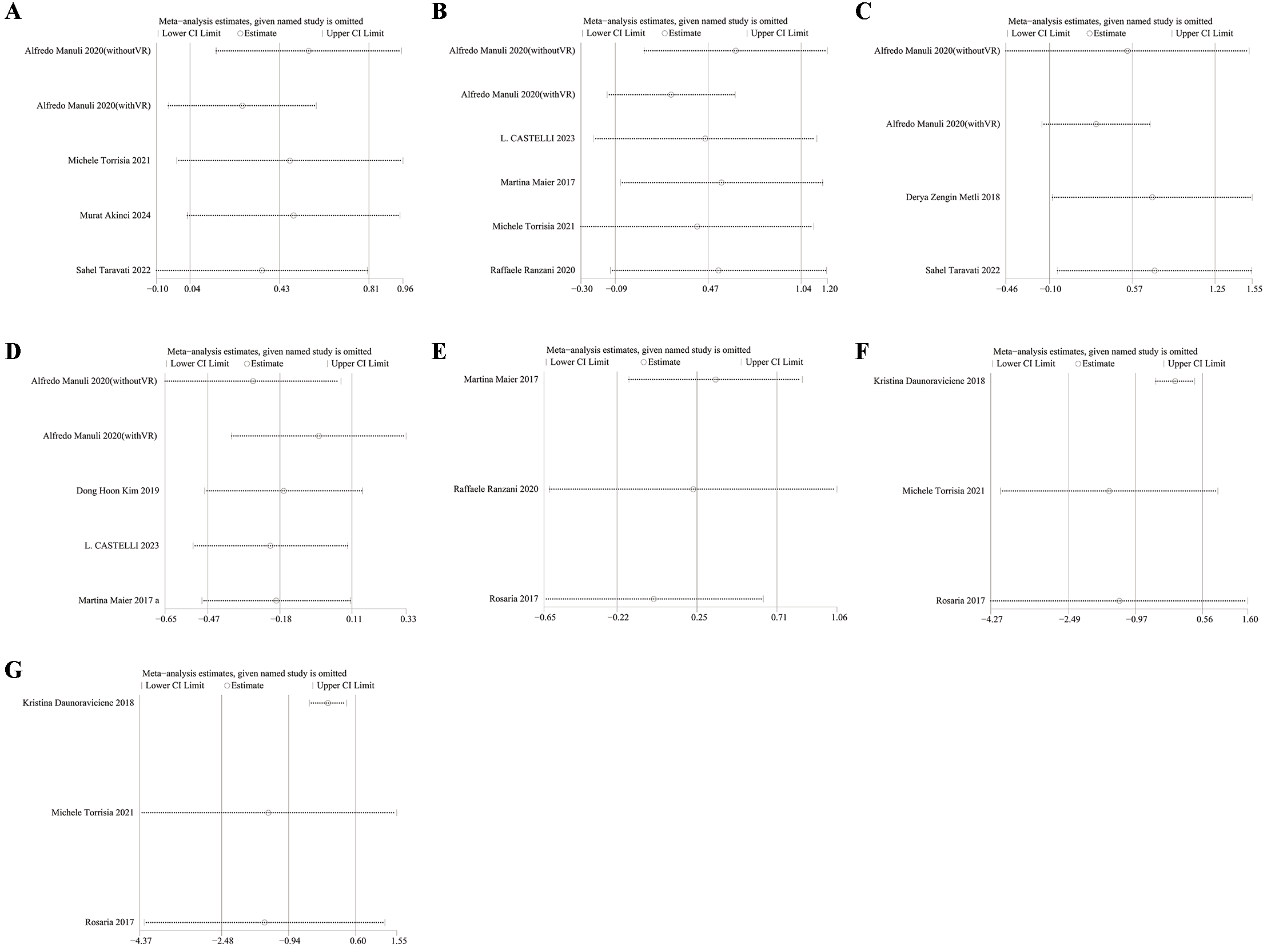


**Figure S13** (A) SEN analysis of the post-intervention MoCA score in the test group vs control group; (B) SEN analysis of the post-intervention FAB score in the test group vs control group; (C) SEN analysis of the post-intervention FIM score in the test group vs control group; (D) SEN analysis of the post-intervention TMT score in the test group vs control group; (E) SEN analysis of the post-intervention MMSE score in the test group vs control group; (F) SEN analysis of the post-intervention HAM-A score in the test group vs control group; (G) SEN analysis of the post-intervention HAM-D score in the test group vs control group.


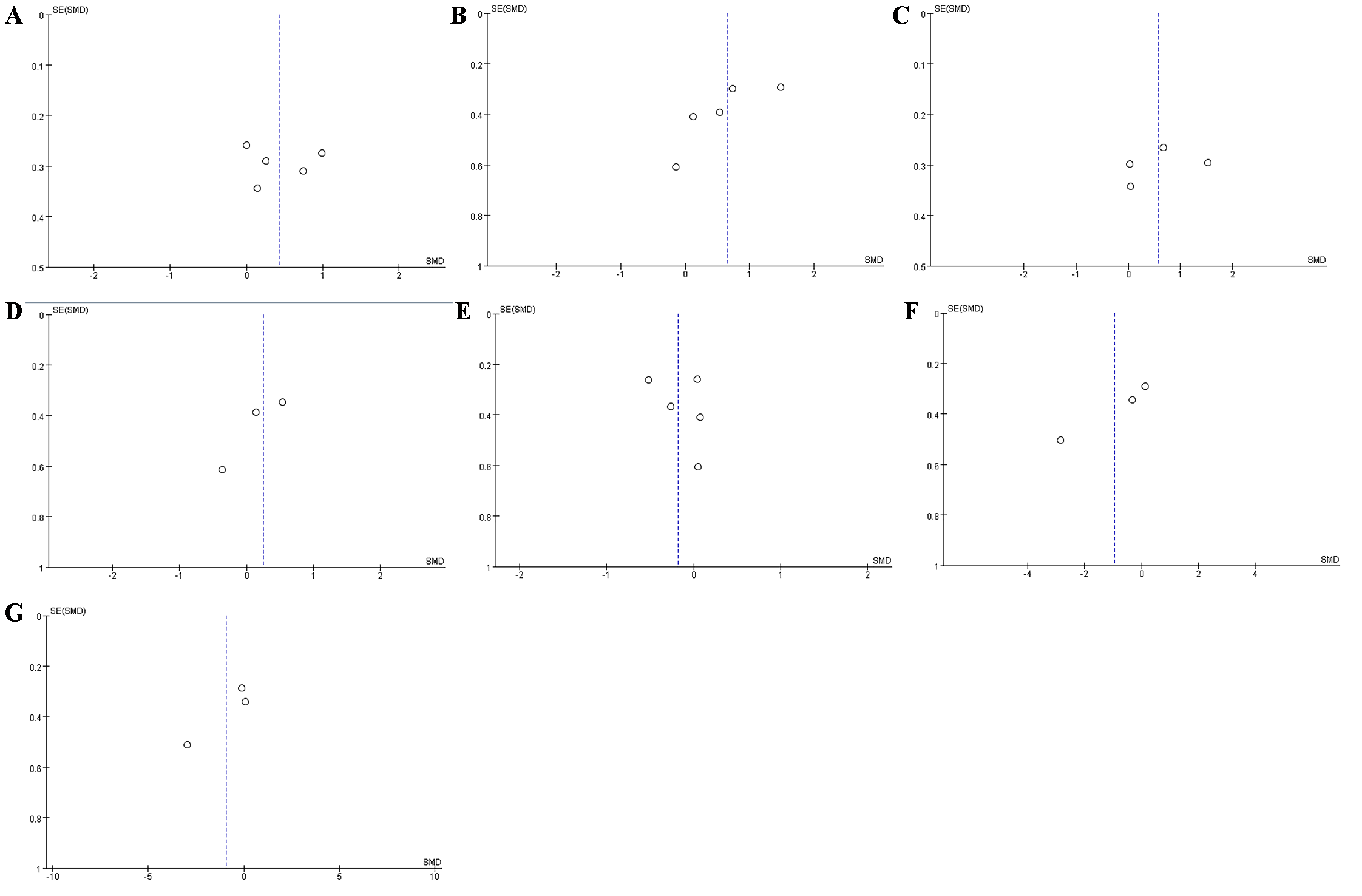


**Figure S14** (A) Funnel plot for the change in MoCA score; (B) Funnel plot for the change in FAB score; (C) Funnel plot for the change in FIM score; (D) Funnel plot for the change in MMSE score; (E) Funnel plot for the change in TMT score; (F) Funnel plot for the change in HAM-A score; (G) Funnel plot for the change in HAM-D score.
